# Increasing important roles of child and adolescent psychiatrists in the treatment of gaming disorder: Current status in Japan

**DOI:** 10.3389/fpsyt.2022.995665

**Published:** 2022-10-19

**Authors:** Masaru Tateno, Takanobu Matsuzaki, Ayumi Takano, Susumu Higuchi

**Affiliations:** ^1^Tokiwa Child Development Center, Tokiwa Hospital, Sapporo, Japan; ^2^Department of Psychiatry, National Hospital Organization Kurihama Medical and Addiction Center, Yokosuka, Japan; ^3^Department of Mental Health and Psychiatric Nursing, Tokyo Medical and Dental University, Tokyo, Japan

**Keywords:** gaming disorder, behavioral addiction, internet gaming disorder, pathological gaming, neurodevelopmental disorder, child and adolescent psychiatry

## Abstract

**Background:**

Digital gaming is the most common leisure activity among children and adolescents in Japan, especially in males. Playing online gaming has become more common among school-age children over the years. As a result, excessive online gaming in younger children has become a significant social problem in Japan. Previous studies have demonstrated that excessive online gaming could cause various mental health issues in children and adolescents. At medical institutions having child and adolescent psychiatry services, there is an increasing number of children and adolescents with various problems related to excessive gaming. The aim of this study was to investigate the current practice of gaming disorder (GD) in clinical settings in Japan.

**Methods:**

The subjects of this study were all of 414 child and adolescent psychiatrists certified by the Japanese Society for Child and Adolescent Psychiatry (JSCAP). The study questionnaire was mailed to all subjects from the official secretariat of JSCAP. Study subjects were requested to answer the questionnaire anonymously. The survey contained three types of responses: open responses; single and multiple-choice responses; and, responses on a five-point Likert scale. The questionnaire consisted of 14 questions regarding GD.

**Results:**

We received 159 responses. The most common reason for a visit to child and adolescent psychiatry service which results in a subsequent diagnosis of GD was school refusal/absenteeism followed by disruption of sleep-awake rhythm. The most common specialized treatment for GD currently offered at child and adolescent psychiatry service is individual psychotherapy. The two most frequently experienced difficulties in the treatment of GD were low motivation to achieve recovery and a large variety of combined problems other than excessive gaming itself. With regard to the three most common psychiatric comorbidities of GD, they were autism spectrum disorder (ASD), attention deficit hyperactivity disorder (ADHD), and depression.

**Discussion:**

The results of our survey revealed that although GD is a behavioral addiction, many children and adolescents with GD first visit child and adolescent psychiatry clinics rather than specialized clinics for addiction which are usually designed and staffed for adult patients. Because it is known that GD is more prevalent among young males, including junior high and high school students, GD has become one of the most important clinical issues in child and adolescent psychiatry today. The important roles of child and adolescent psychiatrists in the treatment of GD has been increasing.

## Introduction

Digital gaming is the most common leisure activity among children and adolescents in Japan, especially in males. A nationwide survey (*n* = 5,096) demonstrated that 85.0% of respondents (92.6% male and 77.4% female) played games at least once in the past 12 months and almost half of all respondents (48.1%) play games mainly online (1, 2). Recently, the age at the beginning of the Internet use has been decreasing. A large scale survey of parents of children under 10-years-old on the Internet use for all purposes (*n* = 2,294) revealed that 33.7% of 10-year-olds, 62.6% of 2-year-olds and 82.3% of 6-year-old children used the Internet for any purposes (3). The results of the same survey also reported that 82.0% of children aged 17 and younger (*n* = 5,805) used the Internet for gaming. A significantly high percentage of elementary school students (*n* = 1,101) have access to the Internet *via* game consoles (72.4%). Although playing online gaming has become more common among school-aged children over the years, Nakayama et al. warned that the results of their survey (*n* = 549) demonstrated that the risk for problematic gaming was positively associated with the younger the age at which weekly gaming begins (4). Taken together, excessive online gaming in younger children has become a significant social problem in Japan. Furthermore, since gaming disorder (GD) was included as a psychiatric disorder in the 11th revision of the International Classification of Diseases (ICD-11) by the WHO (5), the increasing concern about excessive gaming and related problems has been observed among child and adolescent psychiatrists in Japan.

Previous studies have demonstrated that excessive online gaming could cause various mental health problems and be related to neurodevelopmental disorders such as autism spectrum disorder (ASD) and attention deficit hyperactivity disorder (ADHD). A systematic review and meta-analysis by Ostinelli et al., revealed that approximately one-third of individuals with GD also had depression (6). A large-scale cohort in Sweden reported that depression and anxiety were associated with problematic gaming in adolescents (7). Some brain imaging studies suggest that GD and depression may share a common pathophysiology (8). Physical and psychological issues after excessive gaming in adolescents has been reported in Japan too (9, 10). As to neurodevelopmental disorders, a systematic review of 1,028 previously published articles demonstrated that ADHD symptoms were consistently associated with GD (11). A comparative meta-analysis of neuroimaging studies reported the common structural and functional alterations between GD and ADHD (12). There are several other studies suggesting the relationship between GD and ADHD from various viewpoints (13–16). Similarly, there have been several studies on the close relationship between GD and ASD (17–19). With regard to Internet addiction, because young males mainly use the Internet for playing online games, there appears to be a significant correlation with GD. In Japan, a number of studies from clinical practice have been published reporting the association between GD and ASD (20–24). Thus, neurodevelopmental disorders are known to be common comorbidities of GD among children and adolescents.

In Japan, mental health professions have been struggling with long-lasting and unresolved problems associated with the rising number of school refusal/absenteeism cases, many of which apparently increase during adolescence, and often progress to the stage commonly known as “hikikomori,” a condition characterized by severe social withdrawal (25–27). Initially, hikikomori was considered to be a Japanese culture-bound syndrome (28). The Japanese sociocultural background has traditionally been permeated by *amae*, i.e., acceptance of overly dependent behaviors, and shame, which may underlie the similarly culturally bound syndrome called *taijin kyofusho*, a severe form of social phobia (29). However, as more and more papers on hikikomori are published in English, the number of hikikomori cases reported out of Japan has been gradually increasing. Psychological factors that relate to the etiology of hikikomori, such as loneliness, avoidant temperament, introverted personality, and shyness have been reported not only from Japan, but also from other countries (30–32). Each of these psychological factors is also strongly related to Internet addiction (33, 34). The Internet enables individuals with these psychological features to find a normalizing and comfortable place which in turn encourages them to remain there. Recent studies have suggested that hikikomori could at least partially be related to GD (35–38). Because many children and adolescents use the Internet to play online games with both societal and parental approval, it is not surprising that there is a significant relationship between hikikomori and GD where controls are weak and cultural as well as individual predispositions exist.

In Japan, there is an insufficiency of specialized clinics for the treatment of addictions, and where present, they are generally designed for patients over 18 years of age. Moreover, there are very few specialized clinics for treating Internet addiction in Japan (39). Although GD is one of many behavioral addictions, almost all school-aged patients with GD have of necessity first visited child and adolescent psychiatry clinics rather than specialized clinics for addiction. At medical institutions having child and adolescent psychiatry services, there is evidence of an increasing number of children and adolescents presenting with various problems related to excessive gaming such as violence against family members (40), high bills which are consequent to some of the particular games’ rules and rewards systems (41), disturbed sleep-wake rhythm (42), school refusal (35), and various other mental health issues (43). Additionally, a secondary pathway exists through referrals from pediatric clinics, child consultation centers and school nurses for potential diagnoses of GD (40, 44). Nowadays, excessive gaming-related problems are one of the most important issues in child and adolescent psychiatry in Japan. However, no survey has been conducted in Japan on the actual incidence of diagnosis and the status of first-line treatment for GD at medical institutions. All studies on GD, including those from other countries, have been based on self-assessment by either the study subjects themselves or from observation by their family members. To the best of our knowledge, none of the previous studies have asked clinicians to respond to particularized questions relating to their clinical practice in treating GD. This paper does not attempt to delve into the theoretical and/or neurological pathophysiology for GD, nor are the specific forms of treatment a major focus, but rather the related precursory conditions and pathologies, and the relative degrees of incidence are its central themes.

The multi-aims of this study were to survey child and adolescent psychiatrists in Japan who specialize in mental health care of the younger generation with a higher prevalence of GD, in order to: (1) investigate the current practice of GD in clinical settings; (2) understand the most common comorbidities of GD as assessed by child and adolescent psychiatrists; and, (3) ask what challenges are typically faced by child and adolescent psychiatrists in the treatment of GD.

## Materials and methods

### Subjects

The subjects of this study were child and adolescent psychiatrists in Japan. Child and adolescent psychiatry is not an independent specialty and is considered a psychiatric subspecialty here. Instead of a standardized residency program in child and adolescent psychiatry, the Japanese Society for Child and Adolescent Psychiatry (JSCAP),^1^ has its own certification system and the clinician certified by the JSCAP is regarded as a specialist in child and adolescent psychiatry (45). The list of specialists in child and adolescent psychiatry is updated annually on April 1st each year and the number of specialists at the time of this survey was 414. Thus, all of these 414 child and adolescent psychiatrists were invited to contribute to this survey. The subjects were distributed equally throughout the country, but were unevenly concentrated in urban areas due to a shortage of child and adolescent psychiatrists elsewhere. The socioeconomic status of patients seen by each respondent unfortunately could not be collected because of impracticality and privacy issues.

### Study period

This survey was conducted from June 1, 2021 to July 31, 2021. The responses for two respondents who returned theirs after the due date were allowed, and were also included in the analysis.

### Data collection

The study questionnaire was mailed to all subjects from the official secretariat of JSCAP as a postal matter. No additional prompts were made. Study subjects were requested to answer the questionnaire anonymously. The survey contained three types of responses: open responses; single and multiple-choice responses; and, responses on a five-point Likert scale. On the five-point Likert scale, five (5) indicates “Strongly Agree,” three (3) represents a neutral response indicating neither agreement nor disagreement, and one (1) indicates “Strongly Disagree.”

### Study questionnaire

We previously conducted a preliminary survey in Sapporo, Japan to understand the current status and future perspectives of clinical practice for GD among adolescents in January, 2021 (40). In this survey, we used a modified version of the questionnaire which had been used in the preliminary survey. Specifically, we added questions about: (1) whether there is a referable specialized clinic for Internet addiction; (2) common comorbidities of GD; and (3) what challenges are typically faced in the clinical practice of GD.

### Terminology

On the cover page requesting cooperation in the survey, the terms used in this study were clearly defined.

The definition of “gaming disorder” was based on the description in ICD-11 (5) i.e., gaming disorder is characterized by a pattern of persistent or recurrent gaming behavior (“digital gaming” or “video-gaming”), which may be online or offline, manifested by: (1) impaired control over gaming (e.g., onset, frequency, intensity, duration, termination, context); (2) increasing priority given to gaming to the extent that gaming takes precedence over other life interests and daily activities; and (3) continuation or escalation of gaming despite the occurrence of negative consequences. The pattern of gaming behavior may be continuous or episodic and recurrent. The pattern of gaming behavior results in marked distress or significant impairment in personal, family, social, educational, occupational, or other important areas of functioning. The gaming behavior and other features must normally be evident over a period of at least 12 months in order for the diagnosis to be assigned, although the required duration may be shortened if all diagnostic requirements are met and symptoms are severe.

### Questionnaire

The questionnaire consisted of 14 questions divided into seven categories: (1) Questions regarding the respondent’s clinical practice (6 questions); (2) Respondents’ awareness of GD (2 questions); (3) Questions about GD (2 questions); (4) Patient’s chief complaint at initial visit (1 question); (5) Questions about Comorbidity of GD (1 question); (6) Questions about the treatment/intervention provided at the medical institution (1 question); and (7) Questions about the difficulties in the treatment of GD (1 question).

### Ethical issues

This study was approved by the ethics committee of Tokiwa Hospital (TH-21051). The study’s aim was stated on the cover page of the questionnaire sheets that requested the voluntary respondents to answer all questions anonymously. Answering the questions was deemed to constitute consent. The study was conducted according to the Helsinki declaration.

## Results

Out of a total of 414 subjects, we received 159 responses (Response rate: 38.4%). The sociodemographic of the respondents are summarized in Table 1.

**TABLE 1:** Sociodemographic and attitude toward gaming disorder of respondents (*n* = 159).

In which department do they practice? (multiple answers allowed)	*n*	(%)
Child and adolescent psychiatry	139	(87.9)
Psychiatry	93	(58.5)
Pediatrics	2	(1.3)
Psychosomatic medicine	11	(6.9)
Pediatric neurology	0	(0)
Others (descriptive)	0	(0)
**What types of institute do you primarily practice at? (Single answer)**	*n*	(%)
(Child and adolescent) psychiatry clinic (non-hospital)	47	(29.6)
Private psychiatry hospital	36	(22.6)
University hospital	29	(18.2)
Public psychiatry hospital	13	(8.2)
General hospital (without psych beds)	12	(7.5)
General hospital (with psych beds)	11	(6.9)
Child Consultation Center	3	(1.9)
Mental Health and Welfare Center	2	(1.3)
Others (descriptive)	6	(3.8)
**How many years of clinical experience do you have as a physician? (single answer)**	n	(%)
Less than 10 y	6	(3.8)
10 y or more, lee than 20 y	52	(32.7)
20 y or more, less than 30 y	66	(41.5)
30 y or more	35	(22.0)
**What age group(s) of patients do you see? (multiple answers allowed)**	*n*	(%)
Infant/Pre-school	79	(13.3)
Elementary school student	131	(22.1)
Junior high school student	143	(24.1)
High school students and its equivalents	136	(22.9)
Adults	104	(17.5)
**What is the average number of patients you see per month?**	Mean	SD
Patients/month	247.2	225.4
	Median	
	200	
**Is there a specialized clinic of Internet addiction nearby to which you can refer?**	*n*	%
Yes	34	(21.4)
No	124	(78.0)
Blank	1	(0.6)
**To what extent do you agree? “gaming disorder is a psychiatric disorder.”**	Mean	SD
5-point Likert scale (5: Strongly Agree, 4: Somewhat Agree, 3: Neutral, 2: Somewhat Disagree, 1: Strongly Disagree)	3.68	0.92
**Will more patients come to medical institutes in the future with the main complaint of excessive gaming?**	Mean	SD
5-point Likert scale (5: Strongly Agree, 4: Somewhat Agree, 3: Neutral, 2: Somewhat Disagree, 1: Strongly Disagree)	4.26	0.73

SD, Standard Deviation.

With regard to the medical institution where the respondents primarily practice, the most common answer was a clinic (29.6%, *n* = 47). Eleven of the respondents (6.9%) worked at non-medical institutions such as a government Child Consultation Center, a Mental Health and Welfare Center, a juvenile detention center, and a university. 63.5% (*n* = 101) of respondents stated they had more than 20 years of clinical experience.

The average number of patients seen per month was 247.2 ± 225.4 with the median of 200 patients. In Japan, it should be noted that there is a serious shortage of child and adolescent psychiatrists, and each medical institution has a long waiting period to see new patients (45, 46). The results of this survey revealed that each child and adolescent psychiatrist is statistically responsible for the care of a significant number of patients. Although the number of specialized clinics for Internet addiction is small and unevenly distributed as well as concentrated in urban areas, approximately 20% of respondents answered that they had a medical facility to which they could make a referral.

In response to the question regarding child and adolescent psychiatrists’ awareness of GD, 66.0% of respondents agreed that “gaming disorder is a psychiatric disorder.” As to the anticipated needs of medical intervention to adequately address GD, 88.1% of the respondents agreed that predictably more children and adolescents will visit their health care providers in the future with excessive gaming and related problems as their main complaint.

The results and relevant responses to the questions about the current status of clinical practice of GD are summarized in Table 2. The most common reason for a visit to child and adolescent psychiatry service which results in a subsequent diagnosis of GD was school refusal/absenteeism (84.9%) followed by disruption of sleep-awake rhythm (68.6%) which could be recognized as an early sign of school refusal/absenteeism. The most common specialized treatment selected by approximately 30% of the respondents for GD currently offered at child and adolescent psychiatry service was an initial clinical diagnosis and subsequent individual treatment through brief psychotherapy by a child and adolescent psychiatrist.

**TABLE 2:** Current status of clinical practice of gaming disorder (*n* = 159).

How many cases have you seen in the last 12 months with any game-related problems?	Mean	SD
	23.8	67.1
**How many of those cases did you consider to have a gaming disorder?**	Mean	SD
	11.4	42.1
**When a case come to your place with gaming-related problems, what is the main complaint?**	*n*	%
School refusal/absenteeism, absenteeism from work, and frequent tardiness	135	(84.9)
Disturbed sleep-awake rhythm	109	(68.6)
Violence, abusive language, or other violent behavior	93	(58.5)
Patients who were already followed-up for any reason had problems related to gaming and/or internet use	74	(46.5)
Problems related to gaming or internet use itself (excessive gaming/internet overuse, addiction, etc.)	66	(41.5)
Billing problems	45	(28.3)
Referrals from other institutions (including Elementary, Junior high, and High schools, Universities, Child Consultation Centers, Mental Health and Welfare Centers, etc.)	37	(23.3)
Decreased academic performance	32	(20.1)
Somatic symptoms	25	(15.7)
**What kind of treatment do you provide for gaming disorder?**	n	%
Diagnosis and treatment by a child and adolescent psychiatrist	47	(29.6)
Counseling by a clinical psychologist	24	(15.1)
Inpatient recovery programs (hospitalization)	13	(8.2)
Group psychotherapy	7	(4.4)
Family support (family meetings, study groups)	7	(4.4)
Day care (day hospital)	5	(3.1)
**What are the difficulties you experienced in the treatment of gaming disorder?**	*n*	%
The patient does not have awareness of the problem or motivation for treatment	113	(71.1)
The patient has a lot of problems other than gaming/internet use	92	(57.9)
Treatment is difficult and recovery is difficult	78	(49.1)
The patient tends to discontinue coming to the clinic	58	(36.5)
The patient does not come to the clinic	49	(30.8)
Parents do not understand the problem and what parents want is different from what can be provided	44	(27.7)
Long time required for clinical assessment and treatment	28	(17.6)
Difficult to diagnose	26	(16.4)
Requires hospitalization	22	(13.8)

SD, Standard Deviation.

The two most frequently experienced difficulties in the treatment of GD were a lack of self-awareness of the problem in the patients themselves, and low motivation to achieve recovery (71.1%), combined with, and aggravated by a large variety of problems other than excessive gaming itself (57.9%).

With regard to comorbidities of GD, when we asked the subjects to answer what the three most common diagnoses were in order of prevalence, they responded with ASD, ADHD, and depression. The details of answers to this question are summarized in Table 3.

**TABLE 3:** Comorbidities of gaming disorder.

	ASD	ADHD	Depression	Anxiety	OCD	Intellectual disability	Adjustment disorder	School refiusal	Others
First	66	64	8	6	2		1	2	2^a^
Second	61	57	7	10	2	3			
Third	9	13	32	24	9		1	1	6^b^

Gaming disorder often have the same comorbidities and the following diagnoses or symptoms are reported with relatively higher prevalence.

ADHD, ASD, Autism Spectrum Disorder; Depression, Anxiety, OCD, Obsessive-compulsive disorder, Others. In your experienced cases, what are the most frequent diagnoses of comorbidity? In order of frequency, please answer three. (1) Most common [], (2) Second most common [], (3) Third most common [].

^a^Schizophrenia (*n* = 1), Mixed disorders of Conduct and Emotions (*n* = 1).

^b^Blank (*n* = 5), Psychosomatic Disorder (*n* = 1).

ADHD, attention deficit hyperactivity disorder.

## Discussion

The latest version of the ICD, ICD-11, was adopted by the World Health Assembly in 2019 and came into effect on January 1, 2022. One of the most notable changes in ICD-11 is the inclusion of GD as a psychiatric disorder. Accumulating evidence based on various studies including neuro-imaging studies have led to the recognition of GD as a form of behavioral addiction along with gambling disorder (47–49).

The results of our survey revealed that although GD is a behavioral addiction, many children and adolescents with GD first visit child and adolescent psychiatry clinics rather than specialized clinics for addiction which are usually designed and staffed for adult patients. Because it is known that GD is more prevalent among young males, including junior high and high school students, GD has already become one of the most important issues in clinical practice of child and adolescent psychiatry. Our results demonstrated that child and adolescent psychiatrists treated a mean number of 11.4 cases with GD of ICD-11 in the previous 12 months. The number of cases with game-related problems was almost twice as high, and the mean number of cases seen by child and adolescent psychiatrists in the last 12 months was 23.8. Taken together, these statistics indicate that it is not at all uncommon for child and adolescent psychiatrists to be consulted about excessive gaming-related problems at their clinics.

In Japan, Higuchi et al. conducted a large-scale survey on GD including face-to-face semi-structured interviews and developed a nine-item self-rating scale to screen GD, named GAMES test (1). In the study, an average of 5.1% (7.6% in males, 2.5% in females) of the subjects were screened positive to by the GAMES test suggesting that the prevalence of GD in Japan might be close to this number. In our survey, 88% of respondents answered that more children and adolescents, or their family members, were expected to come to their clinics in the future with complaints of excessive gaming and related problems. The demand for medical intervention for children and adolescents with GD will undoubtedly continue to increase.

In Japan, long-lasting school refusal/absenteeism remains an unresolved issue in schools. The rate of students who were absent from school for 30 or more days per year reached 4.1% among junior high school students and 1.0% of elementary school students in 2020 (50). These rates were similar even before the COVID-19 pandemic: 3.9% of junior high and 0.8% of elementary school students were categorized as “school refusal” cases in 2019 (50). In our survey, about 85% of respondents answered that patients who came to their clinic with the chief complaint of school refusal/absenteeism, or frequent tardiness, were later found to have a GD as well. It has been known for some time that long-term school refusal/absenteeism is closely related to hikikomori, a condition characterized by severe social withdrawal (26). The Cabinet Office of Japan conducted a nationwide survey on hikikomori in 2016 which revealed that 540,000 individuals between 15 and 39 years were in hikikomori condition where they remained socially withdrawn for more than 6 months (30). It is quite plausible that GD is one of the factors contributing to and aggravating prolonged school refusal/absenteeism and hikikomori. Perhaps not for all, but at least for some, early detection of, and early intervention for GD might be useful to help these individuals recover from school refusal/absenteeism or hikikomori.

GD often has comorbidities. The results of our survey demonstrated that neurodevelopmental disorders such as ASD and ADHD are the most common comorbidities of GD in the clinical practice of child and adolescent psychiatry in Japan. Regarding the prevalence of ASD in Japan, a Japanese research group demonstrated that the prevalence of ASD was 3.22% in childhood (51). The results of the World Mental Health Japan 2nd Survey reported that the prevalence of ASD was 5.1% and that of ADHD was 8.2% in adulthood (52). When we asked the study subjects to indicate the common comorbidities of GD in their order of prevalence, the most common response was ASD, with ADHD a close second. Approximately 85% of respondents placed ASD and ADHD within the third position. Previous studies have reported that ADHD symptoms were consistently associated with GD (11, 53). Some studies have revealed common findings in the rewards circuit between ADHD and GD (12). Our finding that neurodevelopmental disorders are common comorbidities of GD is consistent with the results of these previous studies including neuroimaging studies. In our study, the third most common comorbidity of GD was depression (29.6%). A survey using Patient Health Questionnaire for Adolescents (PHQ-A) (54) revealed that the prevalence of depression was 13.6% in adolescents in Japan (55). Although the prevalence of depression is higher than the prevalence of ASD and ADHD in the community sample, our results showed that ASD and ADHD were more common comorbidities of GD than depression at child and adolescent psychiatry clinics.

In terms of the treatment, it was found that mainly individual treatments were employed such as brief psychotherapy at outpatient clinics provided by child and adolescent psychiatrists. Presumably, low social skills due to ASD could easily aggravate the patient’s GD, potentially resulting in stiffened resistance to joining group therapy. Only seven of the respondents (4.4%) provided group therapy and only five respondents (3.1%) answered that they had a daycare program for GD, although the clinical usefulness of these programs has been positively reported in Japan (56). Several studies have demonstrated the effectiveness of a therapeutic camp for internet addiction (56–59). In this survey, thirteen respondents (8.2% of the total) answered that they had inpatient programs. Considering that 89 of 159 respondents worked at hospitals with beds, 13% of child and adolescent psychiatrists working at hospitals provided inpatient care for GD. Although therapeutic camps have been reported to be useful for internet addiction including GD, inpatient treatment may be used instead because of the difficulty of organizing camps under the restriction concomitant with the COVID-19 pandemic. On the other hand, because many patients have severe behavioral problems such as irritability and violence, outpatient treatment may be insufficient for pursuing a clinically definable recovery.

Regarding the difficulties the respondent child and adolescent psychiatrists experienced in the treatment of GD, many of them pointed out that patients often do not have a realistic or an adequate awareness of the extent of their problem, or lack substantial motivation to participate in its treatment. Because of the large number of patients assigned to each physician due to a serious shortage of child and adolescent psychiatrists, they may not have enough time—or possibly in some cases the necessary skills—to motivate their patients to be actively involved in the treatment (44). With regard to psychotherapy in the treatment of behavioral addiction, Cognitive Behavioral Therapy (CBT) has been shown to be useful (60). There also is a CBT specially designed for the treatment of Internet addiction (CBT-IA) (61). However, because these psychotherapies were developed for adult cases, they may need to be modified in order to apply them effectively in child and adolescent cases. Since most children and adolescents come to the clinic with their parents, it might be useful to involve their parents in the treatment of GD (62). Our results demonstrated that many patients with GD have comorbid neurodevelopmental disorders. Rather than creating interventions for GD itself, established interventions for comorbid neurodevelopmental disorders may be useful and/or necessary. One avenue for meeting this challenge could be by working in closer collaboration with those psychiatrists specializing in addiction.

This study has several limitations. The sample size was relatively small and the response rate was less than 40%, although we invited all 414 child and adolescent psychiatrists certified by the JSCAP to join us in this study. The differences in sociodemographic between the respondents and non-respondents may have influenced the results. However, it was not practicable to investigate the details of the non-respondents in that vein, nor was an analysis of their patients’ sociodemographic status seen as reasonable in this type of survey. Our subjects did not include psychiatrists working at specialized clinic for addiction because, in Japan, they are customarily seeing only adult patients. The understanding of GD defined in ICD-11 by each respondent, or possibly the lack of it, might also have affected some of the answers to our questions. In this study, we asked child and adolescent psychiatrists about their clinical practice experiences with GD. The authors’ group and other practitioners need and intend to conduct additional surveys addressing children and adolescents with GD in the future. These surveys should be conducted in order to establish effective prevention and treatment strategies for GD.

## Conclusion

To the best of our knowledge, this is the first survey that asked child and adolescent psychiatrists about specific issues in their clinical practice related to GD in Japan. We believe that the results of this survey demonstrate that a significant number of patients with GD as defined by ICD-11 are already seeing child and adolescent psychiatrists, and moreover, GD has become one of the most important clinical issues in child and adolescent psychiatry today.

Children and adolescents with GD often have psychiatric comorbidities such as neurodevelopmental disorders, depression and anxiety. These comorbid psychiatric conditions could be factors that make the recovery from GD more difficult. Furthermore, many child and adolescent psychiatrists are facing various difficulties in the treatment of patients with GD, such as low patient motivation to recover. Also, the challenges of developing a mutually acceptable patient/doctor understanding of what recovery actually means in practical terms, and co-existing multiple behavioral or inextricably related problems apart from excessive gaming, pose further barriers to effective treatment. Our results suggest that clinical guidelines for GD are in order so as to ensure child and adolescent psychiatrists can provide timely standardized, but also individualized, treatment for their patients. Regretfully it cannot be said that an adequate treatment paradigm has been established in spite of growing demands from all parts of the community for effective medical intervention for GD. Further studies are clearly needed.

## Data availability statement

The raw data supporting the conclusions of this article will be made available by the authors, without undue reservation.

## Ethics statement

This study was conducted according to the Helsinki declaration. It was approved by the Ethics Committee of Tokiwa Hospital (TH-21051). The study’s aim was stated on the cover page of the questionnaire sheets that requested the voluntary respondents to answer all questions anonymously. Answering the questions was deemed to constitute consent.

## Author contributions

MT and AT designed the study protocol. MT collected and analyzed the data and drafted the manuscript. TM, AT, and SH reviewed and edited the manuscript. All authors approved the submitted version of the manuscript and contributed to the conception of the study.

## Footnotes

1
https://child-adolesc.jp/
